# Properties of Adenovirus Vectors with Increased Affinity to DSG2 and the Potential Benefits of Oncolytic Approaches and Gene Therapy

**DOI:** 10.3390/v14081835

**Published:** 2022-08-21

**Authors:** Nora A. Bahlmann, Raphael L. Tsoukas, Sebastian Erkens, Hongjie Wang, Franziska Jönsson, Malik Aydin, Ella A. Naumova, André Lieber, Anja Ehrhardt, Wenli Zhang

**Affiliations:** 1 Virology and Microbiology, Center for Biomedical Education and Research (ZBAF), Department of Human Medicine, Faculty of Health, Witten/Herdecke University, 58453 Witten, Germany; 2 Department of Anesthesiology and Intensive Care Medicine, Medical Faculty, University Hospital Cologne, University of Cologne, 50923 Cologne, Germany; 3 Division of Medical Genetics, Department of Medicine, University of Washington, Box 357720, Seattle, WA 98195, USA; 4 Institute of Biochemistry and Molecular Medicine, Center for Biomedical Education and Research (ZBAF), Witten/Herdecke University, 58453 Witten, Germany; 5 Laboratory of Experimental Pediatric Pneumology and Allergology, Department of Human Medicine, Faculty of Health, Witten/Herdecke University, 42283 Wuppertal, Germany; 6 Department of Biological and Material Sciences in Dentistry, Faculty of Health, Witten/Herdecke University, 58455 Witten, Germany

**Keywords:** adenovirus, carcinomas, CD46-detargeting, desmoglein 2, junction opener 4, receptor

## Abstract

Carcinomas are characterized by a widespread upregulation of intercellular junctions that create a barrier to immune response and drug therapy. Desmoglein 2 (DSG2) represents such a junction protein and serves as one adenovirus receptor. Importantly, the interaction between human adenovirus type 3 (Ad3) and DSG2 leads to the shedding of the binding domain followed by a decrease in the junction protein expression and transient tight junction opening. Junction opener 4 (JO-4), a small recombinant protein derived from the Ad3 fiber knob, was previously developed with a higher affinity to DSG2. JO-4 protein has been proven to enhance the effects of antibody therapy and chemotherapy and is now considered for clinical trials. However, the effect of the JO4 mutation in the context of a virus remains insufficiently studied. Therefore, we introduced the JO4 mutation to various adenoviral vectors to explore their infection properties. In the current experimental settings and investigated cell lines, the JO4-containing vectors showed no enhanced transduction compared with their parental vectors in DSG2-high cell lines. Moreover, in DSG2-low cell lines, the JO4 vectors presented a rather weakened effect. Interestingly, DSG2-negative cell line MIA PaCa-2 even showed resistance to JO4 vector infection, possibly due to the negative effect of JO4 mutation on the usage of another Ad3 receptor: CD46. Together, our observations suggest that the JO4 vectors may have an advantage to prevent CD46-mediated sequestration, thereby achieving DSG2-specific transduction.

## 1. Introduction

Carcinomas, malignancies of epithelial tissue, represent the majority of all human cancer cases [1]. The key histological characteristics of epithelial cancers are intercellular junctions that create functional barriers to immune cells, antibody and drug therapies as well as maintaining the tumor micro-environment [2]. There are four main types of intercellular junctions, including adherens junctions, desmosomes, gap junctions and tight junctions [3]. Among these adhesion molecules, desmosomes have been widely recognized and studied for their various roles in cell adhesion, tissue morphogenesis and cell signaling [4]. In detail, desmosomes use cadherin to provide strong adhesion between epithelial cells. There are two families of desmosomal cadherins, desmogleins and desmocollins, that mediate cellular adhesion [3]. In humans, four desmoglein isoforms have been identified. Desmoglein 2 (DSG2) is a calcium-dependent transmembrane adhesion glycoprotein that plays an important role in junction stability [5,6]. Recent studies indicated that DSG2 was downregulated in gastric, prostate and pancreatic adenocarcinomas, which suggested that DSG2 functions as a tumor suppressor [7,8,9]. In contrast, other investigations reported DSG2 upregulation in carcinomas, possibly as a barrier to the immune system and drug therapy. It was demonstrated that DSG2 expression was increased in non-small cell lung cancer, skin carcinomas and human colon adenocarcinomas, implying a possible oncogenic function of DSG2 protein [10,11,12]. In patient-derived melanoma cell lines and tumor tissues, the overexpression of DSG2 was associated with poor clinical outcome primarily because DSG2 can promote vasculogenic mimicry activity in human melanoma cells [13]. These complicated variables underline the need for more research on the role of DSG2 in different carcinomas and their stages of progression. Recently, primary ovarian cancer cells’ DSG2 immunohistochemistry signals and mRNA levels suggested DSG2 as a prognostic and diagnostic biomarker for ovarian cancer [14]. A similar phenomenon was found in patients with multiple myeloma, in which the up-regulated DSG2 on the surface of neoplastic plasma cells was associated with a striking reduction in progression-free survival and overall survival. Moreover, DSG2 directly contributes to the adhesive interactions between multiple myeloma plasma cells and bone marrow endothelial cells, which may support the dissemination of tumor cells to new sites within the bone marrow. Therefore, DSG2 is also suggested as an independent predictor of poor prognosis for patients with multiple myeloma [15].

Adenoviruses have evolved mechanisms to breach epithelial barriers [16]. For example, during the adenovirus type 3 (Ad3) infection of epithelial tumor cells, the fiber interacts with junction protein DSG2 to trigger the transient opening of intercellular junctions [17]. Since DSG2 is a receptor for some species B adenovirus serotypes (Ad3, 7, 11, 14 and 55) [17,18,19], it has a promising role as a target in virotherapy. In epithelial cells, the adenovirus binding of DSG2 triggers events reminiscent of epithelial-to-mesenchymal transition, leading to the transient opening of intercellular junctions. This opening is associated with improved access to receptors such as CAR and Her2/neu that are trapped in intercellular junctions [17,19,20]. An additional characteristic of these serotypes is their production of sub-viral dodecahedral particles (PtDd) during their replication, consisting of Ad fiber and penton base. In the case of Ad3, PtDd are formed at a massive excess (5.5 × 10^6^ PtDd per infectious virus), and it has been hypothesized that PtDd contributes to virus escape and spread [21].

To address the issue of the reduced carcinoma penetration rates of systemically applied oncotherapy, the high-affinity junction opener 4 (JO-4) was developed by mutagenic libraries [22]. JO-4 protein has a single-point mutation at position 239 of the fiber knob amino acid sequence, substituting the native valine to an aspartic acid (V239D), resulting in a nearly 1000-fold increase in affinity to DSG2, as measured in surface plasmon resonance by a shift of the dissociation constant (KD) from 10.8 µM for the original JO-1 protein to 11.4 nM for JO-4. The V239D mutation is localized in a region of the knob corresponding to an exposed loop (E-F). As this region is located at the side of the fiber knob, it is not a direct binding region to DSG2, but it is known to be critical for stabilizing DSG2 binding [23,24]. Notably, only part of the residues critical for Ad3 fiber knob binding to DSG2 are conserved in the other DSG2-interacting adenoviruses (Ad7, Ad11, Ad14 and Ad55). The JO4 mutation (V239D) site is even identified to be one of the two additional residues (VL) that is only present in Ad3, not the other DSG2-using adenoviruses (Ad7, Ad11, Ad14 and Ad55) [22].

The binding of JO-4 protein to DSG2 leads to the shedding of DSG2 followed by a decrease in junction protein expression and transient tight junction opening. JO-4 protein’s synergy with antibody and chemotherapy was proven in efficacy studies with the chemotherapy drug Doxil (doxorubicin liposomal) and ovarian cancer in a mouse xenograft model [25]. Furthermore, preclinical studies proved the safety of this treatment in *Macaca fascicularis* [25]. Currently, the JO-1 protein and its high-affinity version JO-4 protein are under evaluation for clinical application [26,27]. Recent research developed an Ad5-based helper-dependent adenovirus (HDAd) vector with an Ad3 fiber knob containing the JO4 mutation (HDAd5/3+) [28]. This HDAd5/3+ vector can efficiently transduce mobilized hematopoietic stem cells (HSC) in vivo in Rhesus macaques. However, the side-by-side comparison study of JO4 containing virus with its parental virus or with the original Ad3 knob is still lacking.

Previously, other high-affinity fiber knobs from Ad35 were explored to improve the in vitro and in vivo infection properties [29]. For the in vitro studies, high CD46-binding affinity vectors exhibited higher transduction rates in cell lines presenting low CD46 receptor density (K562, MO7e and Ramos suspension cultures). Moreover, the vector with a 60-fold higher CD46-binding affinity was found to be superior in transducing CD46-overexpressing liver metastases after intravenous injection into mice [29]. Considering this fact, adenoviral vectors containing this high-affinity Ad35 fiber knob have been broadly researched for gene and oncolytic therapy [30,31,32].

Our study aimed at producing high-DSG2-affinity viral vectors to improve cell uptake and tumor penetration depth. To this end, we constructed three novel vectors containing the JO4 mutation and investigated them in a panel of tumor cell lines with different DSG2 expression levels to assess these vectors for their infectivity, transgene expression ability and oncolytic effects in direct comparison with their parental viruses. We found that the high-affinity JO4 mutation did not result in correspondingly higher transduction rates of individual viruses. However, we observed that the DSG2 level played a crucial role in the JO4-containing viruses’ activity. In the high-DSG2-expressing cell lines Hs578T and Hela, the JO4-containing viruses displayed comparable transduction levels with their individual parental viruses. In comparison, in the DSG2-negative cell line MIA PaCa-2, the JO4 vectors showed significantly weaker effects than their parental vectors. We further analyzed the effect of JO4 on the usage of CD46 and CAR, another two major adenovirus receptors. CD46 is proven to be a binding and entry receptor for Ad3, especially in the absence of DSG2 [33,34]. Here, we observed a negative effect of JO4 on virus uptake regarding CD46 usage.

## 2. Materials and Methods

### 2.1. Vector Production and Titration

The JO4 mutation was introduced into the adenoviral genome containing plasmids as previously described [35,36,37]. In detail, a PCR-generated selection marker (ccdB-ampicillin) with homologous arms was first inserted into the position to be mutated by linear–circular homologous recombination (LCHR). Then, the selection marker was replaced with a rescue oligonucleotide Jo4-roV239D containing the aimed for T-to-A mutation (for the primers and oligonucleotides used, see Table S1).

To rescue these vectors, the plasmid backbone was removed with pre-inserted restriction enzymes; the released adenovirus genome containing the aimed for modification was transfected into HEK293 cells with calcium–phosphate transfection reagents. After serial passaging to amplify the vectors to large scale, the vectors were purified in two rounds of cesium chloride (CsCl) gradient, as previously described [38]. The adenoviral genome was isolated and sequenced with primer Jo4-seq-f (Table 1) to confirm the aimed for mutation. For titration, vector particles were incubated in SDS-protein K containing Tris-EDTA (TE) buffer and then measured with spectrophotometry at 260 nm as previously described [38].

### 2.2. Cell Cultures

Cell lines Hela, HEK293, Hs 578T, HNC, Huh 7, MDA-MB-231, CHO, Caco-2, MIA PaCa-2 and A549 were cultured in high-glucose Dulbecco’s modified Eagle’s medium (DMEM, PAN-Biotech, Aidenbach, Germany). CHO cells were further supplemented with 1% non-essential amino acid solution (MEM NEAA, 100X, PAN-Biotech). T84 was supplied with half DMEM, half Ham’s F12 (PAN-Biotech). Jurkat and K562 cells were cultured in RPMI 1640 (PAN-Biotech). All the above media were supplemented with 100 units per mL penicillin and 100 µg per mL streptomycin (PAN-Biotech). The Caco-2 medium was supplemented with 20% FBS, while all other media contained 10% FBS from the same supplier (PAN-Biotech). All cells were maintained in a humidified atmosphere at 37 °C and 5% CO_2_.

#### 2.2.1. T84 Cells in Transwell Culture

For the transwell culture experiments, T84 cells were seeded into collagen-coated transwells (24 wells, pore size 0.4 μm, Sarstedt, Nümbrecht, Germany). For this purpose, cells were counted, and 80,000 cells were seeded per well. Cells were cultured in T84 medium (see Section 2.2) from apical and basal sides until confluent, with daily medium changes. After 4–7 days, the apical medium was removed, and cells received a differentiation medium on the basal side for 3 weeks, with 3 medium changes per week (PneumaCult™-ALI Medium, STEMCELLTM Technologies, Vancouver, BC, Canada). According to the provider, this medium can be used for lung epithelial cell culture. Due to the epithelial origin of T84 cells, we applied this medium for the organotypic differentiation of T84 cells in a transwell format.

#### 2.2.2. Spheroid Culture

To form spheroids, approximately 1000 cells per well were seeded of different carcinoma cell lines into U-bottom 96-well plates (Greiner Bio-One, Frickenhausen, Germany) in their respective media. Brightfield and fluorescence images were taken every 12 h using a Satorius Incucyte SX5 live cell imaging system. Numeric evaluation was performed with the Incucyte spheroid software (Incucyte 2021A, Sartorius, Germany).

### 2.3. Analyzing Transduction Efficiencies via GFP Positive Cells

To detect the transduction efficiencies in different tumor cell lines, cells were analyzed with flow cytometry. First, 1 × 10^5^ cells were seeded in 24-well plates. After achieving a complete confluence, cells were infected at various viral particles per cell (vp/c) and incubated overnight. After 24 h, cells were washed once with Dulbecco’s Phosphate Buffered Saline (DPBS) and detached with trypsin-EDTA. Cells were resuspended in DMEM containing 10% FBS, followed by a centrifugation step (1500× *g*, 3 min, room temperature), and washed in DPBS before fixation in 2% paraformaldehyde (PFA). Fluorescence profiles were obtained by analyzing at least 10,000 viable cells on a CytoFlex flow cytometer (Beckman Coulter Life Sciences, Krefeld, Germany). A background signal was obtained by analyzing the negative control consisting of uninfected cells. The percentage of GFP-expressing cells was determined by selecting the region of fluorescence above the background of auto-fluorescence from uninfected cells.

### 2.4. Immunostaining for Receptor Detection

To detect CAR (coxsackievirus and adenovirus receptor) expression on the cell surfaces of different tumor cell lines using flow cytometry, 1 × 10^5^ cells were washed with PBS supplemented with 1% BSA, centrifuged (500× *g*, 3 min) and resuspended in 100 μL PBS/BSA and 1 μL PE-conjugated rabbit anti-hCAR antibody (Antibody Online, ABIN2649016). Following an incubation step at room temperature for 1 h, cells were washed again with PBS/BSA to remove unbound antibodies and resuspended in 100 μL PBS for flow cytometry (Beckman Coulter CytoFlex flow cytometer). The PE-conjugated mouse anti-human CD46 antibody (Thermofisher, Waltham, MA, USA, 12-0469-42) was used to detect the surface expression of CD46 on different cell lines. For the DSG2 detection, Alexa Fluor 488-conjugated mouse anti-human Desmoglein 2 antibody was used (Thermofisher, CSTEM28).

### 2.5. Trans-Epithelial Electrical Resistance (TEER) Measurement of T84 Transwell Culture

The TEER of the T84 cellular barrier was measured in ohm to evaluate the barrier integrity. The transwell culture, which consisted of a multiple layer of cells cultured on a semipermeable filter insert, produced a partition for the inner (or upper) and outer (or lower) chambers. For the electrical measurements, Millicell ERS-2 Voltohmmeter (Merck, Darmstadt, Germany, MERS00002) was applied. Two electrodes, one placed in the inner and one in the outer chamber, were used to measure the resistance of the cellular multilayers.

### 2.6. Histological Staining of T84 Transwell Culture

The histological analysis of the T84-transwell cultures was performed to visually evaluate cell differentiation and integrity. Briefly, the 3D cultures were fixed in 4.5% formaldehyde/PBS. After 24 h of fixation, the round membrane was cut out from the transwell using a sterile and sharp scalpel and padded on blue tissue foam (VWR, F-94216 France). This was subsequently soaked in tap water for 2 h. After dehydration in alcohol, clearing in xylene and embedding in paraffin, the blocks were cut to a thickness of 5 µm with a rotary microtome (purchased from RM 2255, Leica Microsystems, Wetzlar, Germany). Then, the slides were incubated overnight at 37 °C. The blocks were then de-paraffinized at high temperatures, followed by xylene treatment and rehydration with ethanol and washing with deionized water. Subsequently, hematoxylin and eosin staining were performed. The sections were examined under a microscope.

### 2.7. Virus Cellular Entry Measured by Internalization Assay

To quantify the cell entry efficiency, a defined number of adenovirus particles (vp) was used to infect pre-seeded cells in 24-well plates, which were then incubated for different time periods. Cell monolayers were digested and flushed off with trypsin, followed by intensive washing with PBS. Genomic DNA was extracted using Monarch Genomic DNA Purification Kit (NEB, T3010L). To monitor virus genome uptake efficiency, quantitative polymerase chain reaction (qPCR) analysis was performed.

### 2.8. Quantitative PCR Analysis for Adenovirus Genome Quantification

To monitor virus genome uptake efficiency, duplex-qPCR was performed with primers and a probe, detecting the Ad3 fiber knob region (Table S1) to quantify the adenoviral genomes combined with human B2M primers and the probe to quantify cell numbers. QPCR was performed with Probe MasterMix reagent (Takyon, UF-NPMT-B0701) according to the manufacturer’s protocol. PCR cycle was run and detected in the CFX Connect Real-Time PCR Detection System from Bio-Rad (Düsseldorf, Germany).

### 2.9. Oncolytic Assay

Oncolytic assays were performed in 48-well plates. A 3-fold dilution series of individual adenoviruses was freshly prepared to infect pre-seeded cancer cells. The development of any cytopathic effect (CPE) was checked daily until at least one of the viruses on one plate at the lowest dosage showed CPE. The experiment was maintained for a maximum of 14 days. The cells were first fixed with 3.7% formaldehyde, and then the maintained adherent cells were detected by staining the attached cells with 0.5% crystal violet dye. After several wash steps, the stained plates were dried and photographed.

### 2.10. Virus Spreading Ability Detected by Plaque Assay

Plaque assays were performed following a previously published protocol [39]. In detail, cells were seeded in either 10 cm dishes (A549 cells) or 6-well plates (Caco-2 and T84) to achieve a more than 90% confluent monolayer on the day of infection. Each virus was diluted in a 10-fold dilution series and incubated for 1 h with a minimal volume of medium to cover the cell surface. The medium was then replaced with 0.3% agarose in culture medium to form an overlay. This semisolid overlay restricted viral spreading through the liquid growth medium to allow for the formation of discrete countable foci and subsequent plaque formation.

### 2.11. Silver Gel for the Confirmation of Virus Protein Expression

An amount of 1 × 10^9^ vector particles was added to SDS-loading buffer, heated for 5 min at 70 °C and loaded on an 8% separation/5% stacking SDS-gel. For Ad3 and Ad3–JO4, a 4–15% gradient gel Mini-PROTEAN TGX (Bio-Rad, Hercules, CA, USA) was used. Both gels ran at 80 V. The separated vector proteins were stained silver as previously described [40,41].

### 2.12. Statistics

Statistical analyses were conducted with GraphPad Prism 8 (GraphPad Software, San Diego, CA, USA). We used the nonparametric Mann–Whitney U test to identify significant differences between the JO4 vectors and their individual parental vectors (Ad3 vs. Ad3–JO4, Ad5/K3 vs. Ad5/K3-JO4, Ad5/F3 vs. Ad5/F3-JO4).

## 3. Results

The aim of this work was to transfer the previously identified junction opener protein 4 (JO-4) into a viral vector setting. We first generated viral vectors with the JO4 mutation and then explored the effect of different JO4-containing adenoviral vectors in their infectivity, transgene expression ability and oncolytic effect in direct comparison with their individual parental vectors. To test the potential therapeutic benefits of the JO4 variant, we explored different cancer cell lines in two-dimensional (2D) and 3D culture regarding transgene expression level and virus cellular entry. Moreover, to investigate the correlation between virus infection and receptor expression, cell lines with different major adenovirus receptor expression levels were included in this study. Furthermore, these vectors were evaluated in CAR and CD46 knockout A549 cells as well as in stable CD46- and CAR-expressing CHO cells.

### 3.1. Construction of JO4 Containing Vectors

**Figure 1:**
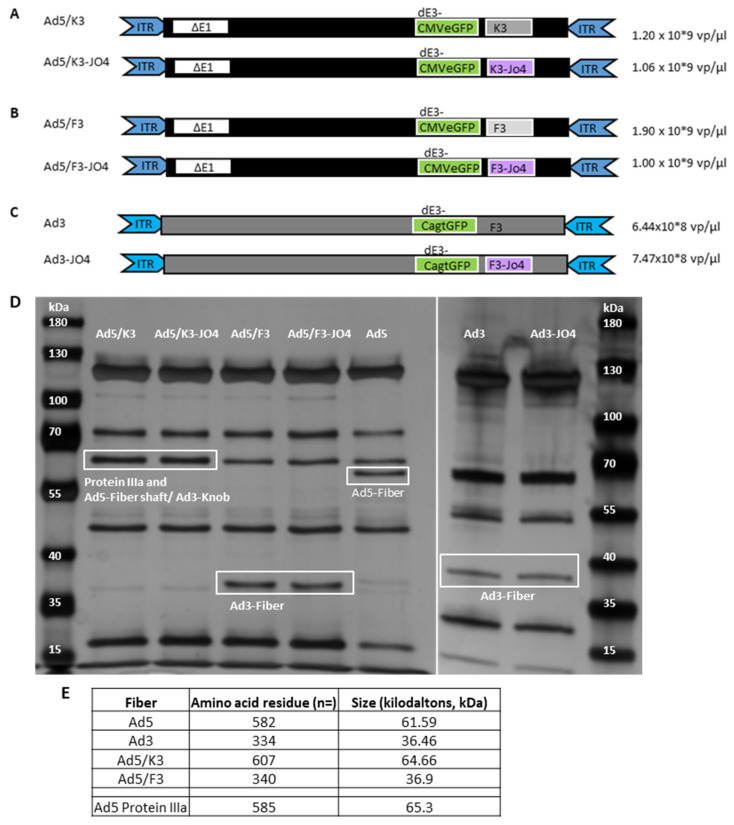
Construction of viral vectors containing the JO4 mutation. The JO4 vectors and parental vectors are presented as follows: (**A**) Ad5-long fiber shaft with Ad3 fiber knob (Ad5/K3) and Ad5/K3-JO4; (**B**) Ad5 with short fiber shaft from Ad3 and Ad3 fiber knob (Ad5/F3) and Ad5/F3-JO4; (**C**) Ad3 and Ad3-JO4 are Ad3-based vectors. All vectors contain a green fluorescent protein (GFP) expression cassette inserted in the E3-region. Ad3 and Ad3-JO4 are replication-competent viruses with the same phenotype as the wild-type virus, while (**A**,**B**) are nonreplicating vectors with an E1 deletion. The physical titers of each virus are shown on the **right**. (**D**) Adenovirus protein detection in silver gel. Fiber proteins are highlighted in labeled rectangles. Note that for Ad5/K3 and Ad5/K3-JO4, the fiber protein is displayed in the same position as Ad5 protein IIIa, demonstrated as relatively strong bands at this position (~65 kDa) compared with Ad5/F3 and Ad5/F3-JO4. The numbers of fiber amino acid residues and sizes are listed in (**E**).

To investigate the affinity-enhancing JO4 mutation in the context of a virus, we constructed three new vectors containing the JO4 mutation (JO4 vectors) (Figure 1A–C). The valine-to-aspartic acid amino acid substitution defined as JO4 in position 239 (V239D) was introduced via previously described linear–circular homologous recombination (LCHR). The first two vectors are chimeric Ad5-based vectors with either an Ad3 fiber, an Ad5 long fiber shaft with only an Ad3 fiber knob (Ad5/K3-JO4) or both a short fiber shaft and a fiber knob from Ad3 (Ad5/F3-JO4). These two vectors are first-generation adenoviruses with a deletion of the early transcriptional E1-region, and thus, they are nonreplicating vectors for potential gene therapy application [42]. Moreover, we generated a JO4-containing vector based on Ad3, which is replication competent. Note that all vectors contain a green fluorescent protein (GFP) expression cassette in place of the early transducing E3-region [36].

To confirm that all JO4 vectors are properly expressing fiber protein, the virus particles were lysated with SDS containing buffer and loaded on silver gel. As shown in Figure 1D, the JO4 vectors display the same protein pattern as their parental vectors regarding protein size and signal intensity. Note that the Ad5/K3 and Ad5/K3-JO4 have the fiber protein and the protein IIIa of similar size at the same position (65 kDa). In Figure 1E is the list of the fiber proteins and the size information on the viruses examined; Ad5 is included as the control.

### 3.2. Transduction Efficiency Measured by Transgene Expression in Human Cancer Cell Lines

**Figure 2:**
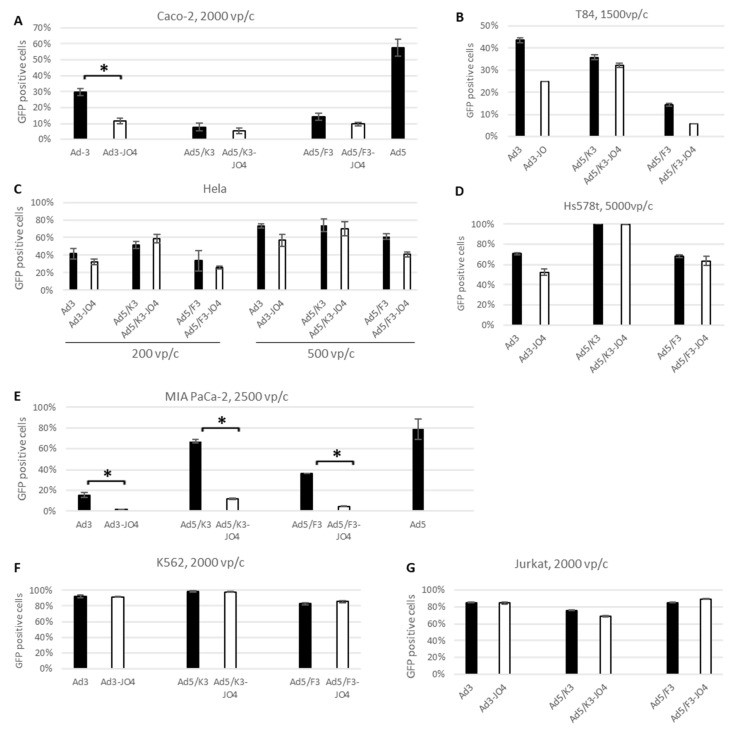
Transgene expression in tumor cell lines with different DSG2 levels. (**A**) Colorectal adenocarcinoma-derived Caco-2 cell line with ~75% DSG2 positive cells. (**B**) Colorectal carcinoma-derived T84 cell line with ~75% DSG2 positive cells. (**C**) Cervical adenocarcinoma-derived Hela cell line with ~100% DSG2. (**D**) Breast carcinoma-derived Hs578T cell line with ~97% DSG2. (**E**) Pancreas carcinoma-derived MIA PaCa-2 cell line with 0.2% DSG2. (**F**) Chronic myeloid leukemia-derived K562 cell line with high DSG2 (85%, Table 1). (**G**) Acute T cell leukemia-derived Jurkat cell line with low DSG2 (41%, Table 1). Cells were infected with the JO4 vectors and their individual parental vectors at the same viral particles per cell (vp/c), and GFP expression levels were analyzed 24 h postinfection with flow cytometry. Uninfected cells (negative control) were used to set the background gate. The percentage provided indicates % of GFP-positive cells. At least 10,000 viable cells were gated for the analysis. Error bars represent standard deviation. (**B**,**D**,**F**,**G**) show one representative of two experiments conducted in technical triplicates. (**A**,**C**,**E**) show one representative of two independent experiments conducted with four technical repeats (* *p* < 0.05).

To explore the effect of JO4 on virus infectivity, the JO4-containing vectors and their parental vectors were investigated in a panel of tumor cell lines. A previous study of CD46 high-affinity Ad35 fiber knob (Ad35++) revealed that the CD46 density played an essential role in the cell line transduction [29]. Therefore, we measured the major adenovirus receptor expression levels and included the cell lines with a broad DSG2 range from below 1% to 100% in the current study (Table 1). The FACS plots showing the CD46, CAR and DSG2 receptor expression levels on the individual cell lines are included in Figure S1. Then, we surveyed the transduction efficiency via transgene (GFP) expression measured in flow cytometry 24 h postinfection (Figure 2).

First, we performed the experiments in monolayer cell culture. Since colon adenocarcinoma-derived Caco-2 and T84 cell lines are frequently used as in vitro models of functional epithelial barriers, we chose these two as models for analyzing the high-affinity DSG2 vectors. The two cell lines showed similar major adenovirus receptor levels (CD46 and CAR) and around 75% DSG2 expression (Table 1). Here we observed comparable transgene expression patterns in the two cell lines after their transduction with respective adenoviral vectors (Figure 2A,B). The JO4 vectors displayed less transgene expression than their parental vectors; Ad3-JO4 in particular showed roughly half as many GFP-positive cells as its parental vector Ad3. An Ad5 control vector transduced Caco-2 twice as efficiently. Moreover, in two high-DSG2 expression cell lines, Hela and Hs578T, all JO4 vectors demonstrated nearly identical transduction levels as their parental vectors (Figure 2C,D). In sharp contrast, the DSG2-negative cell line MIA PaCa-2 demonstrated a significant transgene expression disparity between the JO4 vectors and their parental vectors (Figure 2E). Notably, MIA PaCa-2 was almost resistant to Ad3-JO4 infection at 2500 viral particles per cell (vp/c) with only 1% GFP-positive cells detected, while its counterpart Ad3 showed 15.3% GFP expression. Again, the Ad5 control vector could transduce MIA PaCa-2 efficiently, although it has a low CAR expression.

As many cancers with hematopoietic cell origin show strongly upregulated DSG2 expression and present a potential target for oncolytic viral therapy [15], we also tested the transgene expression of our novel JO4 vectors in K562 (chronic myeloid leukemia) and Jurkat (acute T cell leukemia) cells. In both cell lines, the JO4 vectors resulted in similar levels of GFP-positive cells compared with their individual parental vectors (Figure 2F,G). It seems that in suspension culture, where the virus can interact with cell surface receptors from every direction, the transduction is less DSG2-level dependent.

### 3.3. Virus Spreading in a Two-Dimensional (2D) Cell Culture Model

**Figure 3:**
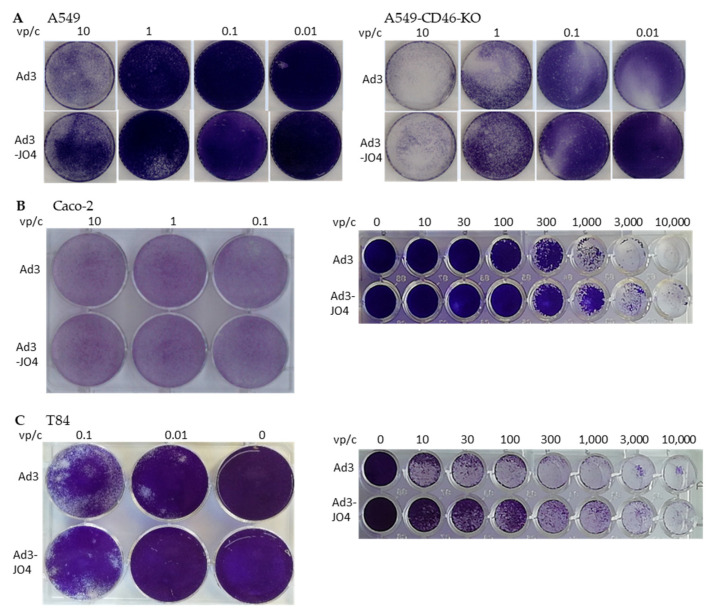
Virus spreading in plaque assay and oncolytic assay. For plaque assays, cells were infected with low doses of Ad3 and Ad3-JO4, and plaques were developed under agarose overlay and stained with crystal violet. (**A**) Plaque assays in A549 and CD46 knockout A549 cell lines. (**B**,**C**) Plaque assays in Caco-2 and T84 cell lines (**left** panel). For oncolytic assays, cells were infected with Ad3 and Ad3-JO4 at ratios shown. Crystal violet staining of viable cells was used to evaluate oncolytic activity two weeks postinfection ((**B**,**C**) **right** panel).

The ability to spread in a tumor is an important feature for the application of oncolytic viruses in solid tumors. Here we investigated the JO4-containing vectors in monolayer cell culture regarding plaque forming and cell lysis (Figure 3).

We first assessed the two replication-competent viruses Ad3 and Ad3-JO4 in A549 cells and in the CD46 knockout A549 cell line (A549-CD46-KO) by applying an agarose overlay to evaluate the virus’s spreading ability. No difference was observed between the two viruses regarding plaque size or number (Figure 3A). It was noteworthy that the viruses displayed a stronger lysis effect in A549-CD46-KO than in A549-WT; this was likely due to the fact that the CD46 knockout generated by CRISPR/Cas9 has certain negative effects on cell growth. Therefore, they are more susceptible to infection and less competent in culture ([43] and data not published). Next, we infected two colorectal carcinoma cell lines, Caco-2 and T84, with Ad3 and Ad3-JO. No distinction was shown in plaque assays, but a three to ten times higher oncolytic effect was observed with Ad3 than with Ad3-JO4 (Figure 3B,C).

### 3.4. Performance of JO4 Containing Vectors in a Three-Dimensional (3D) Cell Culture Model

**Figure 4:**
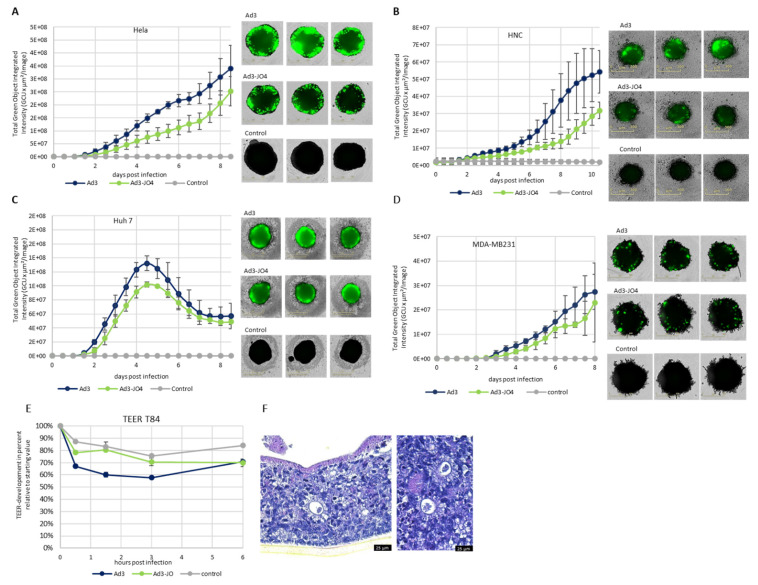
Performance of JO4-containing vectors in 3D cell culture model. (**A**–**D**) Spheroids of human carcinoma cell lines Hela (cervix), HNC (head and neck), Huh 7 (liver) and MDA-MB-231 (breast). Approximately 1000 cells/well were seeded. After four days of culture, dense spheroids had formed and were then infected with 500,000 viral particles/well of Ad3 and Ad3–JO4. (**Left**): development of GFP expression in infected spheroids. Values calculated from green fluorescence images taken every 12 h postinfection. (**Right**): Overlay of brightfield and green fluorescence images 6 days postinfection. (**E**) Trans-epithelial electrical resistance (TEER) measurement of T84-transwell culture after Ad3 and Ad3-JO4 infection. 400 vp/c of both viruses was applied on the apical side (inner chamber). TEER was measured before infection and at 0.5, 1.5, 3 and 6 h postinfection. Values are normalized as percent of TEER (Ohm/cm^2^) relative to starting value, which is defined as 100%. (**F**) Histological analysis of the T84-transwell cultures. HE dye of an apical–basal paraffin cut of T84 cells cultivated in a transwell. There are signs of colon epithelial organotypic differentiation: several crypts that are partially formed by light and mucus-containing goblet cells. For (**E**,**F**), T84 cells were cultured under standard conditions (medium in top and bottom compartments) for 4 days until reaching confluency, and then they were kept under semi-wet interface with a differentiation medium for 25 days until persistent TEER was achieved. (**A**,**B**) represent the means of three independent experiments. (**C**–**E**) represent one of at least two independent experiments. All experiments were conducted in technical triplicates. Error bars represent standard deviations.

Furthermore, we investigated the Ad3-JO4 vector in spheroid culture, which has been used in cancer research as an intermediate model between in vitro cancer cell line cultures and in vivo xenograft tumors. We used a panel of human carcinoma cell lines that were able to grow as dense spheroids and showed high DSG2 expression (over 80% of cells expressed DSG2) including MDA-MB-231 (breast), Hela (cervix), Huh 7 (liver) and HNC (head and neck) cells (Figure 4A–D). An amount of 1000 cells per well was seeded in non-adherent U-bottom 96-well plates and cultivated for 4 days to allow spheroid formation with stable intercellular junctions. The spheroids were then infected with 500,000 viral particles per well of Ad3 or Ad3-JO4. In general, the Ad3-JO4-infected spheroids of all cell lines expressed similar or weaker transgene levels than the ones infected with its parental vector Ad3. Additionally, Ad3-JO4 was not superior in penetrating beneath the surface of the spheroids in this setting.

Since the natural target of Ad3 is polarized epithelial tissue, we then studied the spread of Ad3 and Ad3-JO4 in a colon cancer T84 cell transwell culture model. T84 cells were cultured on semipermeable filter inserts in order to form a multilayer structure with intercellular junctions for four weeks. Amounts of 400 vp/c of Ad3 and Ad3-JO4 were added to the apical chamber. Trans-epithelial electrical resistance (TEER) was measured at the indicated time points (Figure 4E). There was little decrease in TEER observed for Ad3-JO4. To visually evaluate the cell differentiation and integrity, we performed histological analysis of the T84-transwell cultures; colon epithelial organotypic differentiation is evidenced (Figure 4F).

### 3.5. Effect of JO4 on CD46 and CAR Receptor Expressing CHO Cells and Receptor Knockout A549 Cells

**Figure 5:**
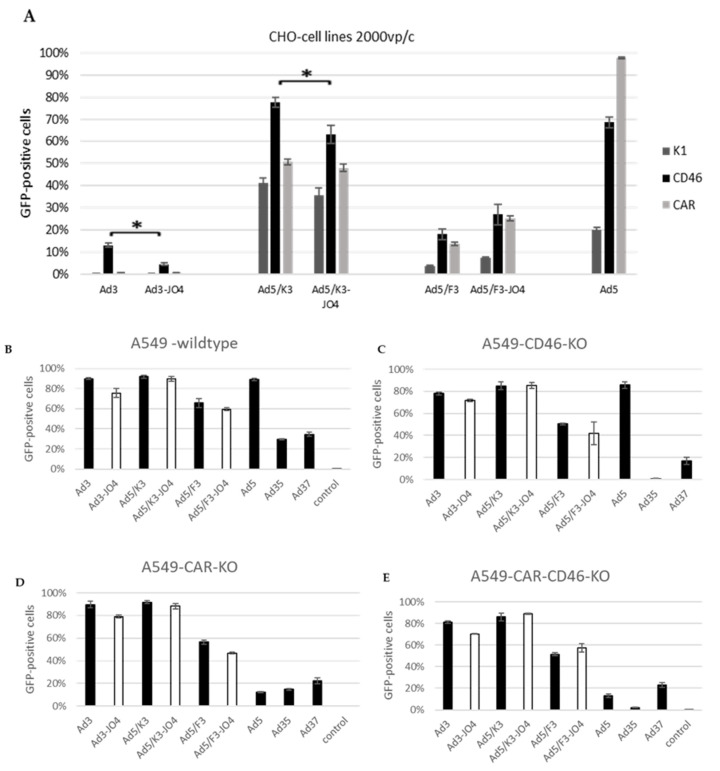
Comparison of transgene expression levels in CHO and A549 cell lines with different receptor profiles. (**A**) CHO-K1, CHO-CD46 and CHO-CAR. (**B**) Wild-type A549 cells, (**C**) CD46-knockout A549 cells, (**D**) CAR-knockout A549 cells and (**E**) CD46-CAR double knockout A549 cells. CHO cells were infected with 2000 vp/c and A549 cells with 1500 vp/c of JO4 vectors and their individual parental vectors. Ad5, Ad35 and Ad37 were included for comparison. Percentages provided indicate the percentage (%) of GFP-positive cells. A total of 10,000 viable cells were counted. All experiments represent one of at least two independent experiments and were conducted with four technical repeats. Error bars represent standard deviation (* *p* < 0.05).

Since we observed a correlation of DSG2 expression level and JO4 vector behavior, we investigated JO4 vectors’ usage of CD46 and CAR. As the CHO-K1 wild-type cell line expresses none of the three primary adenovirus receptors, CD46, CAR or DSG2, and allows neither Ad3 nor Ad3-JO4 to transduce, it is a suitable model for studying the interactions of these two vectors with CD46 or CAR exclusively, using stable CD46- or CAR-expressing CHO cell lines (Figure 5A). The FACS plots showing the CD46, CAR and DSG2 receptor expression levels on the individual cell lines are presented in Figure S2. The transduction of CHO–CD46 cells by Ad3-JO4 was less efficient than that of the parental vector Ad3, and the same was observed on the chimeric vectors with the Ad3 knob (Ad5/K3). In contrast, all the chimeric vectors based on the Ad5 capsid and Ad5 wild-type can transduce CHO-K1. This is evidence for an interaction between the Ad5 capsid and CHO-K1 that permits viral entry independent of primary adenovirus receptors and warrants further study.

Furthermore, we evaluated all three JO4 vectors in comparison with their parental vectors in wild-type A549 cell line, CD46 or CAR knockout and CD46 and CAR double- knockout A549 cell lines (Figure 5B–E). Ad5 was included as a control for CAR expression, Ad35 for CD46 and Ad37 for sialic acid. Sialic acid should be expressed on all four cell lines similarly and therefore should represent a stable marker. Interestingly, JO4 had no perceivable influence on the different vectors’ transduction efficiency. As shown in the supplement (Figure S3), all four cell lines display high DSG2-surface expression levels, and therefore, we presume that the vectors with Ad3-fiber knob are not influenced by the lack of CD46 in the presence of their major receptor DSG2.

### 3.6. Cellular Entry

**Figure 6:**
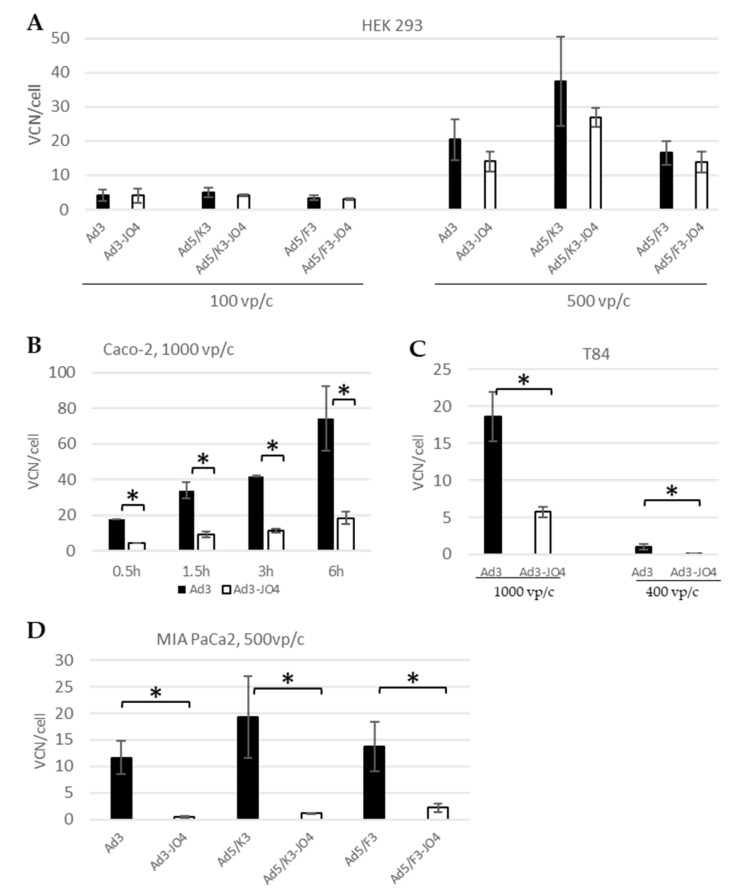
Virus internalization efficiency. Cells were infected with individual viruses at certain viral particles per cell (vp/c) to quantify internalized viral genome copy numbers (VCN), which were quantified by qPCR and expressed as VCN per cell. (**A**) The cell line HEK 293, in which the applied viruses were produced, (**B**) Caco-2 cells, (**C**) T84 cells and (**D**) MIA Paca-2 cells. (**A**,**D**) represent one of at least two independent experiments. (**B**,**C**) were conducted once. All experiments were conducted in four replicates (* *p* < 0.05).

Virus attachment and cell entry are the first steps of infection, and the latter is also used to define the infectious titer or transducing units. Here we quantified the virus genome inside the cells at certain time points postinfection in qPCR (Figure 6). In HEK 293 cells, the cell line in which our viruses were produced, there were no significant differences between the JO4 vectors and their respective parental vectors in viral entry measured as virus genome copy number (VCN) per cell for 500 vp/c and 100 vp/c. In the Caco-2 cells, we monitored the cellular entry with incubation for different time periods and found a time-dependent increase in viral genome from 30 min to 6 h. It is of note that the VCN/cell of Ad3 was four times higher than the VCN/cell of Ad3–JO4. That means that the JO4-containing virus entered the Caco-2 cells with much lower efficiency than its parental virus, which is consistent with the transgene measurement in Figure 2A. A similar phenomenon was observed in the T84 cells, which have the same receptor patterns as Caco-2 (Table 1). The difference in vector cell entry was most apparent for the DSG2-negative cell line MIA PaCa-2. For Ad3–JO4, little virus was detected in the MIA PaCa-2 cells, which confirmed the transduction data (Figure 2E).

## 4. Discussion

Viruses can interact not only with well-displayed receptors at the apical side of epithelial cells but also with poorly accessible receptors hidden in cell to cell junctions [16]. The molecular interaction of human adenovirus type 3 (Ad3) with intercellular junction protein desmoglein 2 (DSG2) has been well studied. During Ad3 infection, the fiber and penton base capsid proteins are produced in vast excess and form hetero-oligomers, called pentons. It has been shown for Ad3 that pentons self-assemble into penton–dodecahedral particles (PtDd) [21,44]. The recombinant Ad3 PtDd, named junction opener protein (JO-1), can bind to DSG2 and trigger intracellular signaling, resulting in the transient opening of junctions between epithelial cells. The opening of tight junctions with JO-1 protein increased the monoclonal antibody and chemotherapy penetration of tumor masses, leading to the greater efficacy of immuno- and chemotherapy [26,45]. Interestingly, not only the co-administration but also the expression of a secreted form of JO-1 protein in Ad5-based oncolytic adenovirus presented a significantly greater antitumor effect than the parental vector [46]. To further increase the therapeutic effect, JO-4 protein, a high-affinity junction opener, was previously generated [22]. Both JO-1 and JO-4 have been shown to be well tolerated in vivo and are being developed as cancer co-therapeutics [26,45].

Notwithstanding its promising application as a recombinant protein, definitive proof of the function of JO4 in the viral life cycle is elusive. Therefore, we generated three adenoviral vectors with the JO4 mutation in the fiber knob. The first two groups are replication-defective chimeric vectors based on E1-deleted Ad5, with either the whole fiber region from Ad3 (F3) or only the fiber knob from Ad3 (K3). The third pair are replication-competent Ad3 vectors. The transduction was first evaluated in a panel of tumor cell lines with DSG2 levels ranging from zero to one hundred percent receptor-positive cells (Figure 2 and Table 1). Surprisingly, none of the JO4 vectors produced any enhanced transduction, in comparison with their parental vectors. On the contrary, the JO4 vectors displayed a rather weakened effect, especially in the low-DSG2 expression cell lines. This was consistent with our measurements of the cellular entry of JO4 vectors and their parental vectors in the same cell lines (Figure 6A–D). In the vector production cell line HEK293, the two virus groups displayed similar cell entry efficiency, while in the two colorectal cancer cell lines Caco-2 and T84, only a quarter of JO4 vectors entered the cells as its control group.

In two-dimensional (2D) submerged culture, we did not observe any difference with plaque-forming assays but saw three- to ten-fold less efficiency of Ad3-JO4 compared with Ad3 in oncolytic assays, suggesting the reduced virus transduction of these tumor cell lines (Figure 3). Since three-dimensional (3D) in vitro models represent important intermediate models between in vitro cancer cell line cultures and in vivo tumors, we next investigated Ad3-JO4 in a panel of carcinoma cell lines in spheroid culture (Figure 4A–D). Similar to the result in the 2D experiment, no enhanced transduction was observed in any of the four different cell lines. In a polarized epithelial tissue sample from the T84 cell line, Ad3-JO4 showed little effect of reducing the trans-epithelial electrical resistance (TEER) (Figure 4E). This could be explained by the fact that DSG2 has high expression on the basal side but low on the apical side. However, the basal side DSG2 is not easily accessible. Due to the JO4 mutation, the virus loses its binding ability to the other major receptor, CD46; therefore, it cannot infect the polarized epithelial tissue efficiently.

Based on the distinguishing features of JO4 viruses in cancer cell lines with different DSG2 levels, we hypothesized that not only DSG2 binding but also interaction with other molecules or the lack thereof played a crucial role in the life cycle of JO4 viruses. In addition to DSG2, CD46 has proven to be a minor receptor for Ad3 infection, and CAR was also hypothesized as such [33,34,47]. To decode the mechanism behind the unexpected weakening effect of the JO4 mutation on the virus, we further explored these viruses in stable CD46- and CAR-expressing CHO cell lines. Since these cell lines express no DSG2 and the wild type is not susceptible to Ad3, the interactions between Ad3 vectors and cells are simply focused on the additional receptors. In CHO-CD46 cells, Ad3 reached 13% GFP expression while Ad3-JO4 reached below 5% (Figure 5A). Moreover, the JO4 vectors and their parental vectors showed the strongest disparity in transgene expression and cell entry in DSG2-negative cell line MIA PaCa-2. The JO4 vectors showed a 6- to 10-fold lower percentage of GFP-positive cells 24 h postinfection than their parental vectors and 6- to 26-fold less VCN/cell 3 h postinfection (Figure 2E and Figure 6D). In contrast with the Ad3-JO4 vector, both Ad5-based vectors (Ad5/F3-JO4 and Ad5/K3-JO4) can infect the DSG2-negative MIA PaCa-2 more efficiently (Figure 2E and Figure 6D). This is probably due to the integrin-mediated cellular entry of Ad5 penton. Although almost all adenoviruses exhibit an integrin-binding RGD motif and require integrin for cell entry, the infection uptake pathways are different [48,49,50,51]. In detail, Ad5 is primarily internalized by dynamin-dependent endocytosis and Ad3 primarily via macropinocytosis. Moreover, the stimulation of infectious macropinocytosis with Ad3 requires both the CD46 receptor and integrin co-receptors. Therefore, Ad3 can use CD46 in combination with integrin to enter the cell; however, we hypothesize that Ad3-JO4 is more limited in that regard due to the reduction of CD46 binding.

We suspect that the valine in position 239 of the fiber knob amino acid sequence serves as a region crucial for Ad3 fiber knob binding to CD46. Moreover, human A549 cells and individual CD46 and CAR knockout cell lines were included. However, here the receptor knockout did not induce a difference in virus transduction between with and without JO4 (Figure 5B–E). We suspect that this is due to the abundance of DSG2 on these cells. It is assumed that Ad3-JO4 has a stronger DSG2 dependency in cell entry than Ad3. This could be a crucial finding for de-targeting the Ad3-based vectors from off-target cells, as CD46 is present on all human nucleated cells. Since DSG2 is not expressed in the mature leukocyte [52], the JO4-containing vectors may have a better chance to de-target peripheral blood cells during systematic application.

A receptor density effect was previously observed in high-affinity Ad35 fiber knob containing viruses (Ad5/35+ and Ad5/35++) [29]. No significantly greater transduction was observed in the different CD46 receptor density-presenting CHO-CD46 cells; the affinity-enhanced viruses only demonstrated higher transduction in low CD46-density human cell line MO7e. Coincidentally, in a measles virus model displaying different affinities of HER2/neu-specific single-chain variable fragments, the experiment on a panel of cells expressing various numbers of HER2/neu receptors also showed that the receptor affinity had little impact on viral attachment [53]. The previous two studies and our present study indicate that the natural evolution of viruses has already achieved intensive selection for variants with an optimal affinity for the infection of their natural target cells. Increasing the affinity to the target receptor made sense when the receptor density was low, e.g., the MO7e cells for the Ad35 fiber knob (35++), which resulted in promising applications in stem cell therapy [32]. In addition to the receptor affinities of the virus knobs, it is broadly accepted that the density and accessibility of the receptors also play an important role in the binding properties of the viral particles to the target cells. Therefore, in the next study, the density and accessibility of the receptors should be evaluated. In our current study, we even observed a negative effect of DSG-binding high-affinity mutation JO4 on virus infectivity, possibly due to reduced CD46 interaction because in DSG2-low/negative cell lines, Ad3 can use CD46 as a receptor, but the JO4 mutation might have affected the CD46-binding region(s) within the Ad3 knob.

Currently, Ad5-based chimeric vectors with fiber knobs from Ad3 and pure Ad3-based vectors represent promising alternatives to traditional Ad5 vectors in terms of receptor usage and preexisting immunity [54,55,56]; in particular, especially the chimeric vector Ad5/3 has already been applied in a clinical trial [57]. The most recent research on in vivo hematopoietic stem cell (HSC) gene therapy achieved around 10-fold higher transduction rates in primitive HSCs in the bone marrow at day 7 after vector injection with helper dependent Ad5/3+ (HDAd5/3+) than the previously used CD46-targeting HDAd5/35++ vector. This might be because HDAd5/3+ prevented CD46-mediated sequestration. [28]. Our work transfers the promising JO-4 fiber protein to the unstudied context of a virus. As a potential in vivo approach, JO4-containing virus may have an advantage in systemic administration for de-targeting CD46 binding, which is especially important for in vivo stem cell therapy.

## 5. Conclusions

In sharp contrast to the efficacy observed with the single JO-4 protein, the JO4 mutation resulted in no enhanced efficiency in the context of a virus, probably due to the negative influence of this mutation on CD46 interaction. Therefore, JO4-containing vectors should be considered in applications to compete with the CD46-mediated sequestration of vector particles in directing DSG2-specific transduction.

## Figures and Tables

**Table 1:** Expression levels of the major adenovirus receptors on the cell surfaces of the cell lines used in this study.

Cell Line	Jurkat	K562	Caco-2	T84	Hs578T	MIA PaCa-2	Hela	A549	HEK293	HCT116	HNC	MDA-MB231	Huh7
CAR (%)	96.4	1.1	93.5	92.6	14.1	8.9	99.7	98.9	97.2	99.5	10.3	84.8	69.5
CD46 (%)	99.4	100	98.5	94.4	99.9	99.8	99.9	99.9	93.5	99.3	99.7	99.8	99.7
DSG2 (%)	41.0	84.5	75.6	74.5	97.4	0.2	99.6	99.8	71.8	90.2	99.3	83.2	94.5

CAR (coxsackievirus and adenovirus receptor); DSG2 (desmoglein-2). Numbers represent percentage of receptor-positive cells in flow cytometry.

## Data Availability

Not applicable.

## Supplementary Materials

The following are available online at https://www.mdpi.com/article/10.3390/v14081835/s1, Table S1. Oligonucleotides used in this study; Figure S1: Analyses of receptor expression levels on different cell lines used in this study; Figure S2: Analyses of receptor expression levels on the used CHO cell lines; Figure S3: Analyses of receptor expression levels on the used A549 cell lines.
