# Supplementary material for: Properties of Adenovirus Vectors with Increased Affinity to DSG2 and the Potential Benefits of Oncolytic Approaches and Gene Therapy
Source: Viruses. 2022 Aug 21;14(8):1835. doi: 10.3390/v14081835 (PMC9412290; doi:10.3390/v14081835)
Supplement: Supplementary file 1 [file viruses-14-01835-s001.zip › viruses-1884925-supplementary.pdf]

Table S1. Oligonucleotides used in this study

Name	Sequence	Note /application
Jo4-ccdB-fwd	TGCCAAGTACTACAGCGTATCCATTTGTTTGTTCAAAAAAAAGCCCGCTC	For JO4 vector construction
Jo4-ccdB-rev	TTTCATTATGTGTTCCCGCATTAGGAAGGCTGACACTCTCTTAAGGTAGC	
Jo4-roV239D*	GCTGACTTTAGTGCAAGAGGTTTTATGCCAAGTACTACAGCGTATCCATTTG**A** CCTTCCTAATGCGGGAACACATAATGAAAATTATATTTTTGGTCAATGCTAC	
Jo4-seq-f	CTATACTTTGATGCCACTGGT	JO4 sequence
FK3-PqP-fwd	TGCCAGATAGTCGCACATC	Viral genome copy number quantification
FK3-probe (FAM)	TGGTCCTTGAATGCTGGTCTAGCTCCAG	
FK3-PqP-rev	GGAGGTTATGAGGGTTGCC	
hB2M-PqP-fwd	GGAATTGATTTGGGAGAGCATC	Housekeeping gene detection
hB2M probe (HEX)	AGTGTGACTGGGCAGATCATCCACCTTC	
hB2M-PqP-rvs	CAGGTCCTGGCTCTACAATTTACTAA	

*The T to A mutation in Jo4-roV239D is highlighted in bold and underlined.

Figure S1: Analyses of receptor expression levels on different cell lines used in this study.

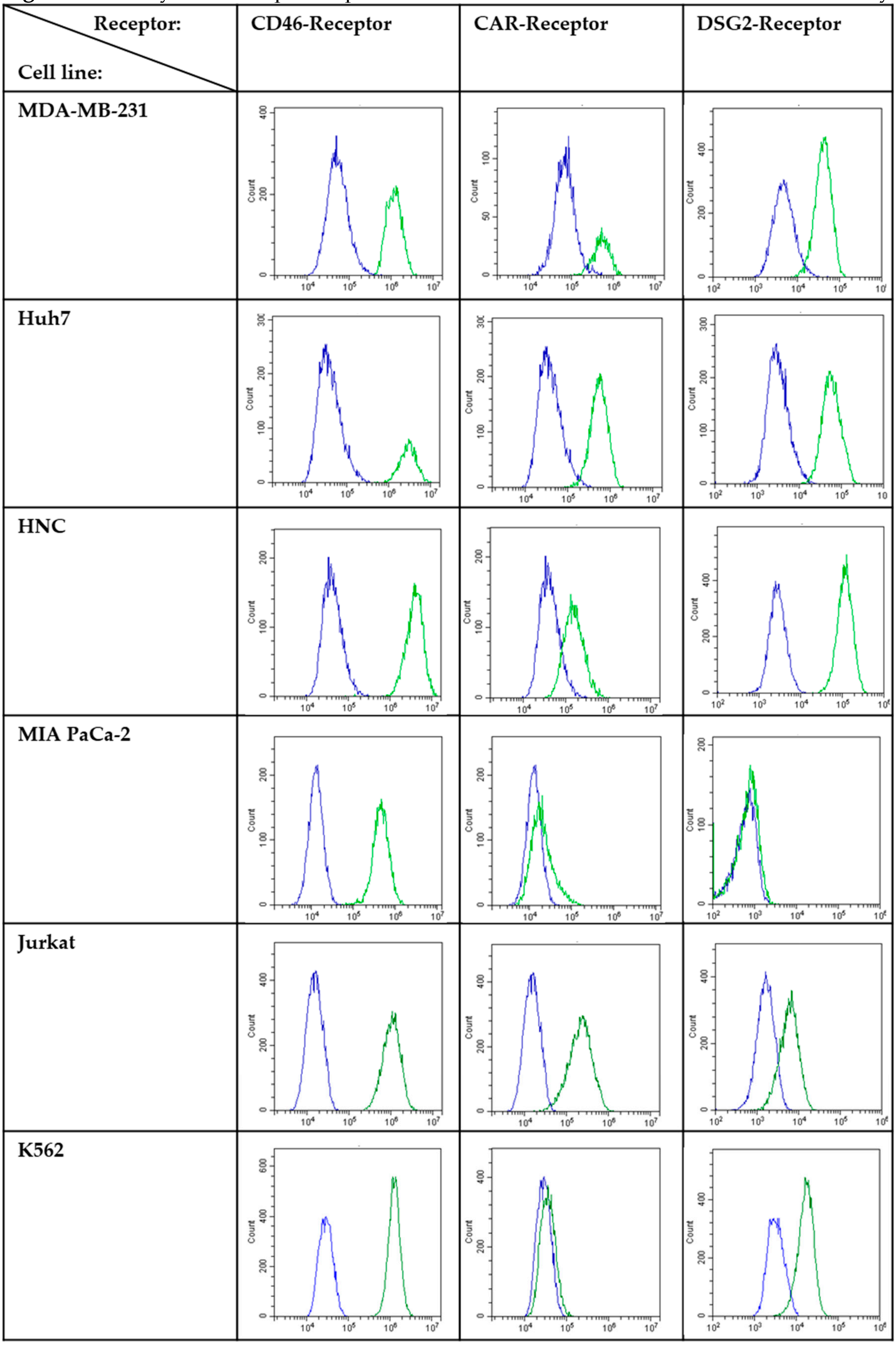

Staining of cell lines with primary adenovirus receptor antibodies (green curve). The blue curve shows unstained cells.

Figure S2: Analyses of receptor expression levels on the used CHO cell lines.

Receptor: / Cell line:	**CD46-Receptor**	**CAR-Receptor**	**DSG2-Receptor**
**CHO-K1**	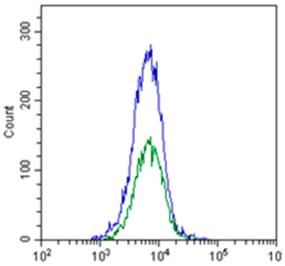	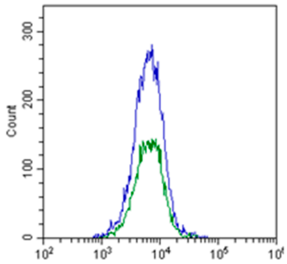	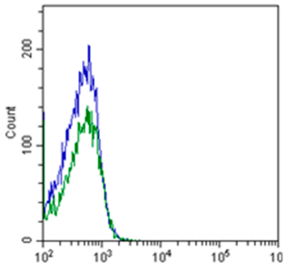
**CHO-CAR**	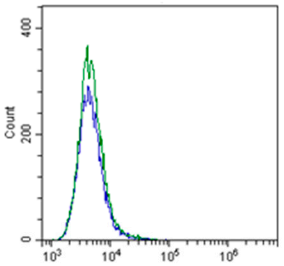	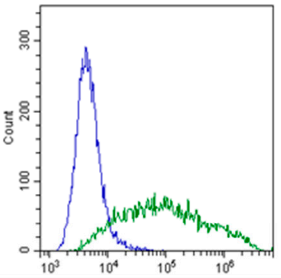	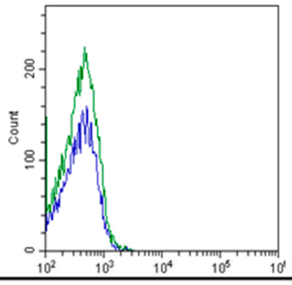
**CHO-CD46**	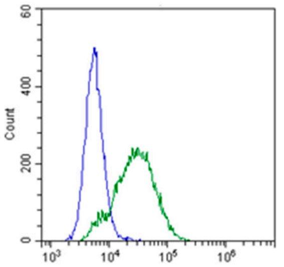	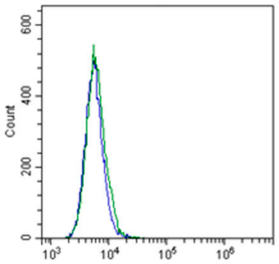	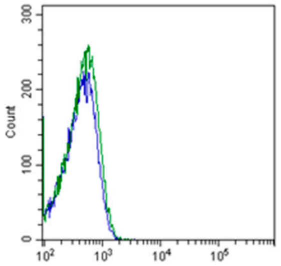

Staining of cell lines with primary adenovirus receptor antibodies (green curve).

The blue curve shows unstained cells.

Figure S3: Analyses of receptor expression levels on the used A549 cell lines.

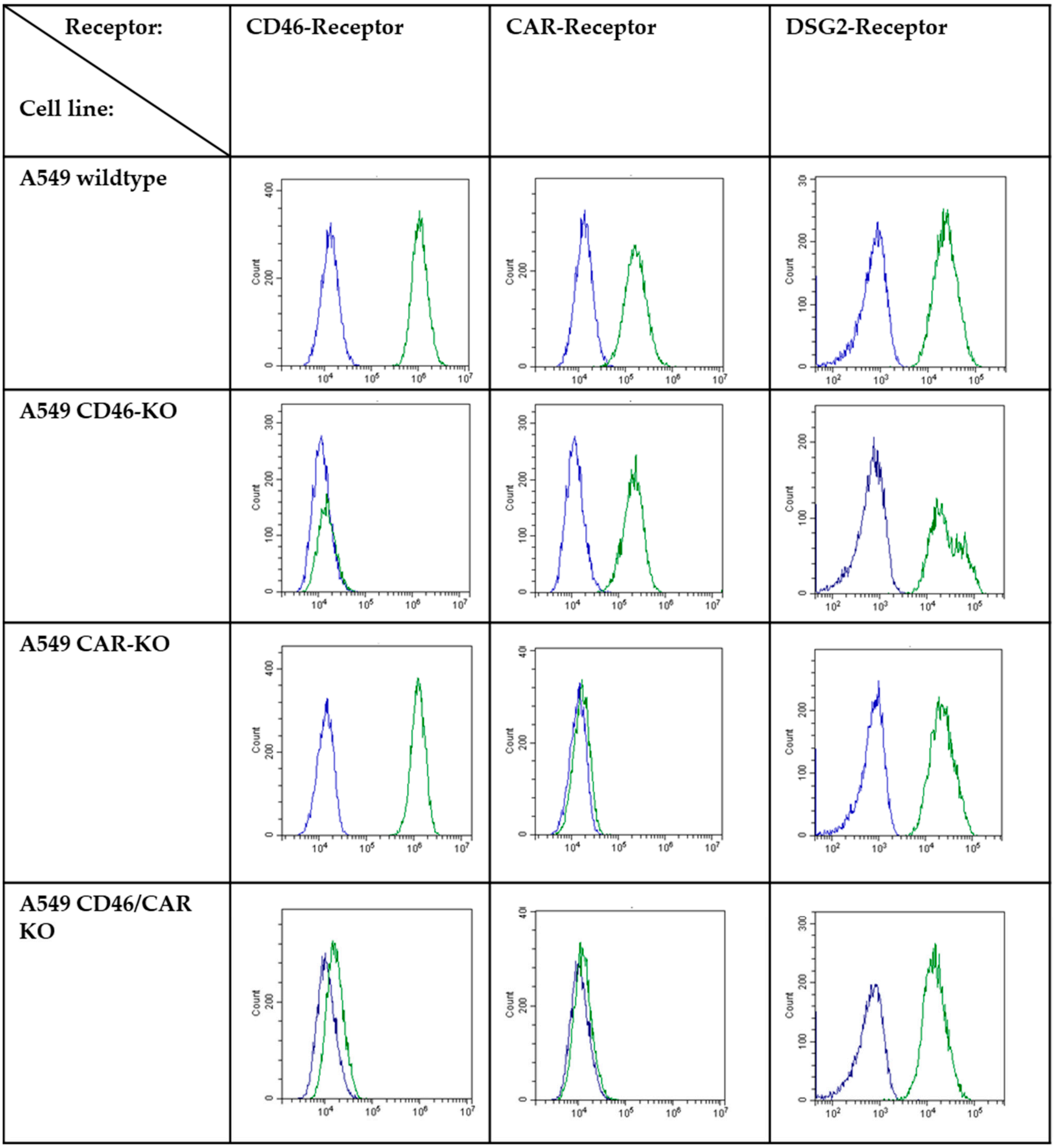

Staining of cell lines with primary adenovirus receptor antibodies (green curve).

The blue curve shows unstained cells.
